# Integrating traditional and allopathic child health: A healthcare transformation opportunity

**DOI:** 10.4102/hsag.v29i0.2501

**Published:** 2024-04-24

**Authors:** Eugene M. Makhavhu

**Affiliations:** 1 Department of Nursing Sciences, School of Healthcare Sciences, Sefako Makgatho Health Sciences University, Tshwane, South Africa

**Keywords:** allopathic healthcare, child health, integration, traditional healthcare, healthcare transformation

## Abstract

**Background:**

Numerous forums both domestically and internationally have discussed integration of allopathic and traditional healthcare. In South Africa, using traditional child healthcare is popular practice. If properly controlled, integrating this aspect of traditional child-health with allopathic healthcare may be advantageous to communities that use both healthcare systems. Allopathic and traditional healthcare remain separate organisations in South Africa despite efforts and discussions to integrate them.

**Aim:**

The study sought to explore the integration of traditional and allopathic child healthcare from the perspectives of children’s caregivers and traditional healthcare practitioners.

**Setting:**

The study was conducted in a semi-urban area in the city of Tshwane.

**Methods:**

An exploratory qualitative research study was conducted using semi-structured interviews to collect data from 11 traditional healthcare practitioners and 15 children’s caregivers who were sampled using snowball and convenient sampling respectively.

**Results:**

The participants expressed their understanding of the usage of traditional healthcare practitioners in the care of children as well as their support for integration, and further indicated its necessity. Religion and its effects in health-seeking behaviour were cited as a factor in why there has not been greater integration between the two healthcare systems.

**Conclusion:**

The undocumented and undisclosed use of traditional healthcare potentially hinders the delivery of therapeutic healthcare. As such, integrating the two systems is essential to ensure patients’ safety.

**Contribution:**

This article highlights understanding of culture congruence and safe child healthcare that may be brought forward by the integration of the two healthcare systems.

## Introduction

*The South African Traditional Health Practitioner’s Act No. 22 of 2007* defines traditional healthcare practices as the performance of a function, activity, process or service based on a traditional philosophy that includes the utilisation of traditional medicine or traditional practices (Republic of South Africa 2007). The Act further indicates the objects of traditional health practices as the maintenance or restoration, diagnosis and treatment of physical or mental illness or the rehabilitation of a person to enable resumption of normal functioning within family or community or the physical or mental preparation of an individual for puberty, adulthood, pregnancy, childbirth and death (Republic of South Africa 2007).

A traditional health practitioner is a person registered under the Act in any of the recognised categories (Republic of South Africa 2007). There are various categories of traditional healers in South Africa, including *sangomas* [diviners], herbalists, traditional surgeons, traditional midwives and spiritual healers (Zuma et al. 2016). The *sangoma* is the category of practitioner most prevalent in South Africa where practitioners are trained under initiation processes when they respond to their ‘ancestral calling’. They have a role in patient outcomes and are widely used for child healthcare needs within South African communities. Some of the uses in child healthcare include traditional child strengthening, naming ceremonies and protecting children against illnesses (Makhavhu, Masala-Chokwe & Ramukumba 2023).

Both traditional and allopathic healthcare practices are common in South Africa, and at times patients would seek the assistance of a traditional health practitioner before presenting themselves to a medical doctor or a healthcare clinic. For many people around the world, traditional healthcare and medicines are the first port of call, and traditional healthcare practitioners have played an important role in maintaining their health and treating chronic illnesses (World Health Organization 2023). However, it is not unusual for patients to seek healthcare interventions from both healthcare practices to cater for their health needs. Traditional health practitioners are frequently the first to interact with patients and are more accessible to them than allopathic medical practitioners in many contexts (Trimble & Rajaraman 2017). It is for that reason that integration of these practices may be beneficial and has been a point of discussion in different forums locally and internationally.

Integration is defined as the action of successfully combining or mixing two or more things (Cambridge English Dictionary n.d.). Integration is further seen as a system that draws connections between previously differentiated phenomena and finding a novel, simplified and higher-order perspective on how they can be related (Rintamäki & Saarijarvi 2021). The British colonial rule and apartheid government in South Africa played a role in prohibiting open access to traditional healthcare under the pretext of witchcraft, and this led to secret consultation with traditional health practitioners (Molebatsi, Breed & Stafford 2020), which continues to date and plays a role in obstructing the integration of the two healthcare practices.

Integrating the two healthcare practices may improve accessibility of healthcare facilities in communities and may also afford an opportunity to address different healthcare needs in child health. Thus far, the only successful, regulated and functional integration between the two practices is through the *Application of Health Standards in Traditional Circumcision Act, No. 66 of 2001* (The Province of the Eastern Cape 2001). However, mainstream healthcare including child health is not catered for despite the continued use of such practices by children’s caregivers.

This happens despite the fact that the *South African Traditional Health Practitioners Act 22 of 2007* recognises traditional healthcare practices and practitioners. Owing to poor regulation of traditional healthcare practices, there seems to be a failure of successfully integrating the two practices. This affects patients who are users of both healthcare practices as they would often not disclose the use of traditional healthcare practices because of the anticipated responses and actions of their allopathic healthcare practitioners once disclosed.

Many reasons may be brought forward to support the continued use of traditional healthcare practices in communities. Some of the reasons for the continued use of traditional health practices may include the affordability of traditional medicine, the perceived inefficacy of biomedical and/or allopathic treatments, the effectiveness of traditional medicines and patients’ perceived incurability of diseases with biomedical or allopathic healthcare (Legesse & Babanto 2023). About 55% of people in South Africa live in poverty (World Bank Group 2020) and most are unable to access healthcare with only 16.4% of the population in 2018 being covered by medical aid (Government Communication and Information System 2019) to access private healthcare. Other people live far from public health facilities and thus need transport to access them (Makhavhu et al. 2023), whereas traditional healthcare practitioners are easily accessible and familiar to the communities that may have used them for generations. These factors make the integration of traditional and allopathic child healthcare vital and thus necessary to be explored further in order to enhance safe, effective, holistic and accessible child healthcare services. Therefore, the aim of this study was to explore the perspectives of children’s caregivers and traditional healthcare practitioners towards the integration of traditional and allopathic healthcare methods in a semi-urban area in the City of Tshwane, South Africa.

## Research methods and design

### Study design

A qualitative exploratory research design was utilised in this study. Qualitative studies describe the experiences of life and culture of participants (Gray, Grove & Sutherland 2017), and exploratory studies are those used to describe perspectives of participants related to a specific problem (Grove & Gray 2021). This qualitative exploratory research design was used to explore the integration of practices from the traditional healthcare practitioners and the children’s caregivers as providers and users of healthcare, respectively, and therefore a qualitative exploratory approach was appropriate.

### Study setting

This study was conducted in a semi-urban area of Soshanguve, which is located about 37 km north of the city in the Tshwane Metropolitan Municipality, Gauteng province in South Africa. The township is equipped with lower levels of healthcare at the primary healthcare level including a 24-h community healthcare centre and seven clinics that operate from 07:00 to 16:00. The facilities offer preventative and curative care including child health care while the community health centre further offers emergency services and maternity care in the maternity and obstetric unit (MOU) on a 24 h basis. There is a sound distribution of traditional healthcare practitioners of different categories within the community including *sangomas* [diviners], herbalists, traditional birth attendants and traditional surgeons. They offer different services as needed by community members, and they are often visible by their distinct items of clothing and beads that are unique to them. Traditional healthcare practitioners are further distinguished in the communities by their *indumba*, which is a sacred healing space for the ancestors (Rogerson 2017), which is used by traditional healthcare practitioners to consult their clients and is often a hut located on the side or behind their main houses.

### Population and sampling

The population in this study included 26 participants of which 15 were children’s caregivers and 11 were traditional healthcare practitioners. In this study, children’s caregivers referred to any one person who was from the township of Soshanguve and was a guardian or a parent, either biologically or not, and had the authority to make healthcare decisions about the child’s health. Traditional healthcare practitioners in this study referred to any practitioner regardless of their category who was fully initiated was actively practising as a traditional healthcare practitioner and had experience working with children as their patients. Any participant who did not meet those criteria was excluded from the study. Snowball sampling (Gray et al. 2017) was used to sample traditional healthcare practitioners, whereas children’s caregivers were sampled using convenience sampling (Brink & Van Rensburg 2022). The sample size was 26, and this was determined by saturation among the two populations when no new information emerged (Brink, Van der Walt & Van Rensburg 2018).

### Data collection

Data were collected from a total of 26 participants using a semi-structured interview guide. The author conducted individual interviews with children’s caregivers in two clinics in the community, while the traditional healthcare practitioners were interviewed individually in locations pre-arranged with them. For some participants who could understand and respond adequately, interviews were held in English. For those who could not understand English but could grasp and respond appropriately in Setswana, interviews were conducted in that language as it is one widely spoken in the township. The interviews were audio recorded using a tape recorder, and the participants were made aware of and gave permission for the recordings. Interviews took between 30 and 45 min, which was dependent on the participants’ responses to questions also considering the two populations. The question posed to the participants was ‘What are your perceptions regarding the integration of traditional and allopathic healthcare practices for child health?’ Based on the participants’ responses to the main question, further probing questions were asked.

### Data analysis

Content analysis (Gray et al. 2017) was used to analyse the data. Interviews were audio recorded and transcribed verbatim by the author. The transcripts were read in order to deduce and code recurrent statements from the participants’ responses that were then categorised as themes. Emergent themes from transcripts were coded and to corroborate the conclusions and the recurring themes that appeared during data analysis, an independent coder’s assistance was requested. With the consultation between the coder and the author, a consensus discussion materialised and three themes were agreed upon.

### Trustworthiness

To ensure trustworthiness in this study, principles of credibility, dependability, confirmability and transferability as described by Lincoln and Guba (1985) were adhered to. Participants were interviewed in a language they could understand, and the criteria for participation were clearly laid out for participants. To ensure credibility, the study used triangulation of data sets where two different populations were asked questions about the same phenomenon. Member checking was applied where the researcher used paraphrasing and probing during interviews to capture participants’ meaning, and some questions were re-visited to obtain the exact meaning. To ensure dependability of the study, an audit trail of data and other records has been kept for auditing purposes. All interview transcripts and audio records are kept safe and accessible only to the researcher. To ensure confirmability, the researcher ensured that data collected accurately reflect the responses as narrated by participants and applied bracketing to avoid biases.

### Ethical considerations

The study followed ethical principles of research including autonomy, informed consent, privacy and confidentiality, justice, beneficence and non-maleficence (Brink & Van Rensburg 2022). Informed written consent was obtained from participants, and they were made aware of their rights to self-determination and that they could cease participation if uncomfortable to continue. Interviews were conducted at participants’ convenience. Confidentiality was ensured as conducted interviews were coded by number, and no identifiers were used in the research reports. All participants were treated fairly during the study period. The study received ethical clearance from the university [FCRE 2020/05/03 (FCPS 02)(SCI)] and the district health authority [GP_202006_002], which was granted prior to the commencement of data collection.

## Results

### Demographic data

The study included 26 participants in total. Representation was split between 11 traditional healthcare practitioners (THPs) and 15 children’s caregivers. All participants were black, of African descent and aged between 19 years and 58 years. A total of four male participants were represented, while 22 participants were female. Of the male participants, two represented traditional healthcare practitioners, while the other two were children’s caregivers. Of the 22 female participants, nine represented traditional healthcare practitioners, and 13 participants were children’s caregivers.

### Overview of themes

Through analysis of data, three themes were identified, namely: Perceptions on positive outcomes of integration, barriers to integration and fostering integration (Table 1).

**TABLE 1:** Themes and sub-themes.

Theme		Sub-theme
1.	Perceptions on positive outcomes of integration	1.1 Integration between the two healthcare systems supported 1.2 Necessity and importance of integration 1.3 Multiple healthcare choices for patients
2.	Barriers to integration	2.1 Religion 2.2 Political resistance 2.3 Lack of scientific evidence to support traditional health practice 2.4 Lack of trust in traditional health practice 2.5 Poor regulation of traditional healthcare practices
3.	Fostering integration of allopathic and traditional child healthcare	3.1 Communication 3.2 Further research on traditional healthcare practices 3.3 Stakeholder engagement

*Source:* Makhavhu, E.M., 2023, ‘Development of strategies to facilitate integration of indigenous-traditional and allopathic child health services in Soshanguve’, Doctoral thesis, Tshwane University of Technology

### Theme 1: Perceptions on positive outcomes of integration

Participants emphasised the value of and expressed support for integration between the two healthcare systems. There was gratitude voiced regarding integration, which suggested that integration would call for the exploration of healthcare choices to support patients’ healthcare-seeking behaviours. According to participants, improved communication between both healthcare practitioners would result from integration. Some of the statements made by participants are shown in the quotes below:

‘I would feel very good if the two would work together. At least I would have an option in healthcare and they will be working together to help patients and they will as well learn the different methods from each other.’ (Caregiver 6, female, 22 years old)

‘It would be really good to integrate. At least I would know that if a doctor can’t help me, then they will tell me where to go. I mean I can’t come to a clinic every day and the only thing they give me is Brufen and a rub [*methylsalicylate muscle rub ointment*]. At least if they couldn’t help, then they can refer me.’ (Caregiver 7, female, 34 years old)

‘Well because at the end I will be seeking help, I would take my child to where I am told there is help. Whether it is the hospital, clinic, church, or a traditional healer. Because at that time all I would want is for my child to be healthy and healed from whatever it is they are suffering from so I really would be okay with the integration. I would support the integration and not have any problems.’ (Caregiver 11, female, 22 years old)

Participants continued to emphasise the potential, necessity and importance of integration. It was suggested that integration might result in positive patient outcomes for clients of both healthcare practices. Furthermore, other participants indicated knowing the strengths and effectiveness of traditional healthcare practices and traditional herbs and concoctions. Participants’ quotes are indicated below:

‘I think it is right, and very important because if a person comes to the clinic and get treatment, I believe sometimes our bodies still need the spiritual healing and protection. Most traditional herbs are very helpful and they build strength in our bodies. Personally, I know they work. I have been through a really difficult time where I was very sick, I was taking my treatments from the hospitals but it wasn’t enough. But since I started making use of the herbs, I saw myself picking up and getting much better.’ (Caregiver 15, female, 35 years old)

‘Yes, I think so [*that integration is important*]. There are things that as healers we need from the hospitals and some that they need from us. We both have our weaknesses and strengths that may benefit the patients if we could work together.’ (THP9, female, 39 years old)

Additionally, participants also noted other conditions thought to be only treatable by traditional methods. They further noted referral practices between the two healthcare systems as another method of integration and building a relationship between the two. Some of the responses from participants are highlighted in quotes below:

‘I think integration is possible and necessary, I think for example if a child has “hlogwana” [*sunken fontanelle*] and the clinic gives the child a drip, I can then put together the herbs to heal the child completely and we would have worked together there.’ (THP6, female, 58 years old)

‘It is necessary to work together, and yes, it is possible mkhulu, like if I am helping a patient, and I refer to a hospital. They can maintain contact and call me when it is difficult and they don’t see what to do. That way, we would build a relationship between the two of us and I may be able to tell them that we should do this and not that and they can do the same with me.’ (THP7, female, 46 years old)

In contrast, traditional healthcare practitioners were more interested in how integration would help both healthcare systems. They stood out particularly for their strong support of the referral component of patient care, whereas caregivers in this study focused more on the advantages of integration in terms of providing healthcare consumers with options regarding their healthcare requirements.

### Theme 2: Barriers to integration

The participants also mentioned their perceived barriers to the two healthcare systems’ integration. Most participants mentioned religion as a barrier to the integration of the two healthcare systems in South Africa:

‘People are now deeply rooted in religion and think that everything else that is not is evil. Others grew up in households where African spirituality was practised but they decide to drop when they grow older and they say they are saved and born again.’ (Caregiver 4, female, 20 years old)

‘It is just the belief and people thinking that the use of traditional medicines is demonic and ungodly.’ (THP10, male, 47 years old)

‘I think there is too much interference from religion. People say that because they go to a certain church, they believe only in God and that they do not believe in the use of traditional health practices or rituals. So they will rather go to a clinic or a hospital rather than a traditional healer.’ (Caregiver 10, female, 28 years old)

‘Those ones who say they are bazwalane (Christians). They will make it difficult to integrate. They are those that think that indigenous-traditional healthcare practitioners are witches and that they practise witchcraft, so if we can take that out of the window as well then maybe it could work better.’ (Caregiver 5, female, 50 years old)

It was further indicated that the two healthcare systems are currently isolated because of people’s beliefs and church sermons that discourage the use of traditional healthcare practices and that some people usually refer to the use of traditional healthcare practices as demonic, ungodly and associated with practicing witchcraft. This was a common finding in both traditional healthcare practitioners and the caregivers:

‘I think the people that refer to themselves as “bazalwane” [*Christians*] and working in the hospitals and clinic will not agree because they always say they have no business with sangoma because it is against their beliefs. They are brainwashed by modern and white systems to believe that our systems are evil and ungodly.’ (THP3, female, 36 years old)

‘I think it has a lot to do with belief more than anything else. People, especially doctors and nurses in the hospitals don’t believe that our herbs work. They don’t believe that you can dig up something from the ground, mix it with others and sometimes maybe even cook it and then give it to a person as medicine. Traditional healers feel that they are not respected as they do not have access to help the people that need their services. Even from the people who say they are Christian and do not do things in a certain way.’ (THP1, female, 46 years old)

‘It will be very difficult because people will say we are practicing witchcraft when we go consult [*a traditional healthcare practitioner*].’ (Caregiver 6, female, 22 years old)

The participants also brought up the lack of scientific support for traditional healthcare practices and methods, as well as the absence of political will as a barrier to integration. Quotes below highlight the statements as raised by participants:

‘I think if the government was white, there would have been integration already. So I think it’s not happening because of the lack of action from the government. And at the same time our beliefs are different so there would be more suspicion of witchcraft. So the debate around it is too much so it will be difficult to integrate because of the beliefs we have as black people. So yeah. There is too much political resistance.’ (Caregiver 12, female, 38 years old)

‘Well, traditional healers use herbs. They are not formally manufactured. They are found at the mountains and not tested in the lab which makes people to doubt them and their effectiveness.’ (Caregiver 15, female, 35 years old)

Another barrier to integrating the two healthcare systems as noted by participants was the lack of confidence and respect in traditional healthcare practices and their treatment methods. Other participants felt that it might be risky to have the two systems of healthcare working together because of the lack of knowledge of treatments used. The majority of the blame for the disrespect towards traditional healthcare practices was placed on allopathic healthcare practitioners:

‘Usually, just because of beliefs and lack of respect. I mean if I am coming from school for seven years, I probably would not take anything from someone who went to initiation for three months or less on how to treat a patient.’ (Caregiver 13, female, 21 years old)

‘I think there is no respect towards our system of traditional health and we know a lot of things.’ (THP4, female, 26 years old)

‘I think the allopathic healthcare professionals, whether black or white, do not believe in our systems and our herbs. They don’t think our medicines work. They don’t think anything we do works.’ (THP6, female, 58 years old)

‘Okay. I think it’s very risky as you do not know what the child is being put into. You don’t know what danger it poses to the child or what substances were used in that muthi [*medicinal herbs and concoctions*].’ (Caregiver 8, female, 23 years old)

Another participant made reference to poor regulation of traditional health practices as well as the lack of collegial relations among traditional healthcare practitioners as another constraint to integration. Quotes below highlight the response:

‘I think amongst the traditional healers, there is no togetherness and working together. The gazette for traditional healer’s regulations was drafted a long time ago neh, but they can’t regulate us because the indigenous-traditional health practitioners are not agreeing with a lot of things. They are pulling in different directions. Others do not want to be regulated and want to do their own things uncontrolled. They know that immediately we are regulated, the practice will be standard and we will know that this is how one must treat this and that. So if regulation can happen, then maybe we can be able to integrate well.’ (THP11, male, 50 years old)

Participants in this study, both caregivers and traditional healthcare practitioners, had similar sentiments regarding the role of religion as a barrier to integrating the two systems of healthcare.

### Theme 3: Fostering integration of allopathic and traditional child healthcare

In order to close the gap and allow integration between the two healthcare practices, communication and understanding between practitioners from the two practices were highlighted as crucial factors to take into account. Other participants thought that by talking about patient consultation, treatments and their consequences with stakeholders, the two practices of healthcare may become more understandable, and the gap could be closed, further indicating a need for training to allow engagement into the practices to improve knowledge of traditional health practices:

‘I think communication. People who work in clinics and hospitals must be able to communicate about these kind of things as well. So there should be communication on how they could help each other and how that would benefit the patients so then they will be able to understand each other better.’ (Caregiver 6, female, 22 years old)

‘I think communication between the two systems and training each other on their ways and practices so that they may understand better what the others do.’ (Caregiver 9, female, 35 years old)

‘I think if we can all meet up with the hospitals and clinics and others [*stakeholders*] and speak about this issue and show each other that this is what we can do and here is what we may not be able to do so that we can understand each other’s practices.’ (THP2, female, 57 years old)

‘I think they need to sit down together, get the perspectives and discuss ideas so they can teach each other and both understand what the others do to treat their patients and how one can learn from the other.’ (Caregiver 11, female, 22 years old)

Some of the participants indicated a need for further research into traditional healthcare practices, their treatment modalities and herbs in order to advance traditional health practices comprehension. Others emphasised the necessity of registering traditional healthcare practitioners in hospitals so that they can be reached in cases of emergencies or when patients make requests of them:

‘So I would really suggest further research in such medicines and if they approve it then people can go there if comfortable to use such. Until then, I feel they are not really safe.’ (Caregiver 1, female, 27 years old)

‘Hmmmm, that will be a difficult thing to try. The thing is nurses and doctors need to put themselves in the shoes of the healers ‘cos I think they are the ones who are most likely to refuse to work with them. Yeah, So if they were to leave the disrespect and put a bit of trust in this systems as well. I just think they must find a way.’ (Caregiver 3, male, 40 years old)

Another participant indicated keeping a register of local traditional health practitioners who can be linked up to a clinic in order to assist where patients’ treatment is deemed to have failed to respond to allopathic healthcare systems and where the patients may feel the need to consult traditionally:

‘I think at the hospitals, they can maybe register traditional healers and keep our numbers so they can be able to contact us if there is a problem they have dealt with by all their means and failed or they believe it requires intervention of healers. I think then we can work well together, or even if they just give the numbers to the patient and say the patient can call us and come see us.’ (THP5, female, 36 years old)

In contrast, traditional healthcare practitioners were more inclined to focus on the aspects of communication that other caregivers had also brought up, while caregivers on the other hand appeared to be in favour of researching traditional healthcare practices in order to increase the likelihood of safe integration.

## Discussion

This study sought to explore the perspectives of children’s caregivers and traditional healthcare practitioners towards the integration of traditional and allopathic healthcare methods in child health services in a semi-urban area of the City of Tshwane Metropolitan Municipality, Gauteng province in South Africa. The study provides an insight into the much talked about phenomenon of integration between two systems of healthcare in a country where both are accessible, but practicing in silos. The accessibility of healthcare would ideally make an impact on people’s health-seeking behaviour while in the emerging world’s population, there is about 80% of reliance on traditional healthcare practices and medicines for therapy, which includes child health. It is reported that about 80% of the black South African population seek healthcare from traditional healers (Mothibe & Sibanda 2019), whereas in other African countries such as Ghana, more than 80% of patients were found to have consulted a traditional health practitioner first before they went to a hospital (Nolna et al. 2020 as cited by Boum et al. 2021). The use of African traditional healthcare and medicines plays a large role in the management of different conditions including chronic management and preventative, curative and palliative healthcare, and this is done in a holistic approach (Mothibe & Sibanda 2019). Such use warrants a successful and workable integration between the two systems of healthcare systems in order to continuously deliver safe patient care.

Traditional healthcare practitioners in this study indicated that they supported the integration of the two systems and that it would benefit patients if it was done correctly. This finding was in line with Green and Colucci (2020), which suggested that traditional health practitioners and allopathic healthcare practitioners recognised that integration between the two would benefit patients. Children’s caregivers were also mainly supporting the integration; however, some were sceptical about the risks that may be involved with integrating the two.

Despite the majority of participants claiming support for the integration of the two healthcare systems, they raised what they perceived as barriers to implementing the integration. This study discovered that one of the integration-restraining elements was Christianity. It is believed that the presence of Christian missionaries in Africa was responsible for the introduction of Christianity to South Africa, which in turn contributed to the advancement of modern medicine. Christianity was introduced into South Africa by European settlers and later missionaries in the 17th century (Mokwena 2021). Modern medicine was therefore more accepted than traditional medicines, and traditional health practitioners were gradually frowned upon by individuals of the Christian faith as found in this study. There was a similar finding in Mokgobi (2016). Prior to the advent of Christianity, people of South Africa made use of traditional healthcare practices, the same as in other neighbouring countries such as Botswana where the arrival of the Dutch Reformed, Seventh Day Adventist and Lutheran churches introduced modern medicine to the country (Togarasei, Mmolai & Kealotswe 2016). Participants further indicated that some people of the Christian faith perceived the use of traditional healthcare practices as witchcraft and as well referred to them as being ungodly. This more frequently framed around people of the so-called ‘charismatic churches’ or the Pentecostal churches in South Africa, which are known as ‘Bazalwane’ or the ‘Born again’ Christians. This finding is supported by a study by Nemutandani, Hendrick and Mulaudzi (2020) who found that the introduction of Euro-western culture, practice and religious beliefs, such as the Christian faith, dominated and disregarded the indigenous knowledge systems.

In addition to religion, this study discovered that some participants believed the absence of a scientific foundation for traditional healthcare practices could also be perceived as a barrier to integration. The claim of a lack of scientific basis was made in reference to the fact that traditional health practitioners treat their patients using herbs rather than officially tested drugs like those used in allopathic medicine. A study in Botswana also found that participants referred to clinics and hospitals as having ‘proper’ medications and diagnosis criterion that are scientifically proven and administered in ‘right’ dosages (Togarasei et al. 2016). This lack of testing may make some patients to be sceptic about using traditional healthcare practitioners citing safety reasons, whereas allopathic healthcare practitioners may as well exhibit the same attitude of questioning patients’ safety associated with the use of traditional healthcare practices. This finding is supported by a review study conducted by Green and Colucci (2020), which presented multiple authors’ findings that biomedical practitioners’ concerns were primarily rooted in patient safety and human rights. These worries might be well founded because, in certain cases, traditional healthcare practitioners act unethically, endangering patient safety in the absence of regulation. Another study by Oseni and Shannon (2020) found that there was a problem with accepting the spiritual aspect of traditional health practitioners among participants. Participants in this study also indicated that there was a lack of respect and negative attitudes towards traditional healthcare practices, and this was similarly found in Van Rooyen et al. (2017) who found that the lack of referral practices from allopathic to traditional healthcare practices was because allopathic health practitioners had a negative attitude towards traditional health practitioners.

Successful regulation as indicated in the *Traditional Health Practitioners Act No. 22 of 2007* (Republic of South Africa 2007) would encourage registration of traditional practitioners; however, there is currently poor regulation of traditional practices, which also makes it difficult to know and follow up on who is practising. This study found the lack of regulation of traditional healthcare practitioners as another barrier to integration as reported by some participants. This is also as reported by Keikelame and Swartz (2015) that the lack of regulation was a barrier to effective collaboration.

This study also discussed ways to enable integration more effectively. Participants mostly discussed creating communication and stakeholder engagement platforms to discuss services provided and consequences of patients in order to encourage integration by being aware of these issues. Other participants suggested that patient referrals between healthcare professionals in the two systems of healthcare could serve as a starting point for fostering integration. A study by Musyimi et al. (2016) also suggested capacity building as well as a referral system to co-manage patients as a strong factor to promote integration between the two systems of healthcare. This study also suggested that further research be done in order to understand more about traditional health practices and their associated medicines in order to foster integration and trust.

### Limitations of the study

Traditional healthcare practitioners often view their practices as sacred and are often timid to disclose some aspects of their practices. As a result, this affects the quality and the depth of data that could have been collected.

## Conclusion

This study extends knowledge about the child healthcare from the perspectives of caregivers and traditional healthcare practices. It is important to note that traditional healing methods and plants are widely employed in South Africa, much like in the rest of Africa, and that it is expected that this trend will continue for a variety of reasons. This article demonstrated the hindrances to integrating the two systems of healthcare and the continued undocumented use of traditional healthcare practices owing to such hindrances. Healthcare delivery should be inclusive and take into account various communities, cultural backgrounds and health-seeking behaviours. Therefore, in order to strengthen integration, healthcare regulations should be strengthened for traditional health in the same way that they are for allopathic healthcare.
